# Editorial: Advanced molecular targets in the diagnosis and treatment of gastrointestinal cancers, volume II

**DOI:** 10.3389/fonc.2024.1528137

**Published:** 2024-12-03

**Authors:** Zsolt Kovacs, Cornelia Braicu, Simona Gurzu

**Affiliations:** ^1^ Department of Biochemistry, Environmental Chemistry, “George Email Palade” University of Medicine, Pharmacy, Science and Technology of Targu Mures, Targu Mures, Romania; ^2^ Centre of Research in Oncology and Translational Medicine, Centre of Research in Oncology and Translational Medicine (CCOMT), Targu Mures, Romania; ^3^ Centre of Research for Functional Genomics, Biomedicine and Translational Medicine, University of Medicine and Pharmacy Iuliu Hatieganu, Cluj Napoca, Romania; ^4^ Department of Pathology, “George Email Palade” University of Medicine, Pharmacy, Science and Technology of Targu Mures, Targu Mures, Romania; ^5^ Department of Medicine, Romania Academy of Science, Bucharest, Romania

**Keywords:** gastrointestinal cancer (GI cancer), targeted therapy, personalized treatment, precision medicine, molecular oncology

**Figure 1:**
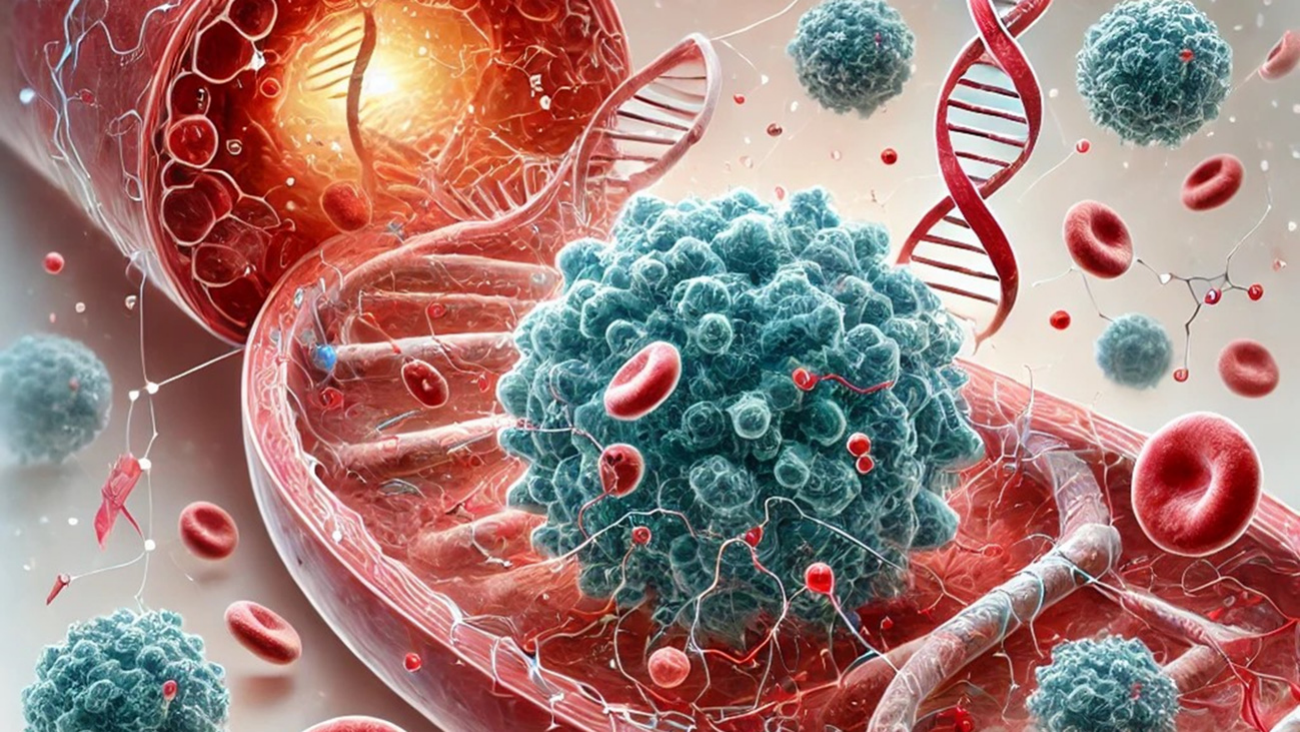
Graphical representation of the Research Topic.

In our Research Topic entitled “*Advanced Molecular Targets in the Diagnosis and Treatment of Gastrointestinal Cancers, volume II*”, we managed to publish 15 articles, original researches, case studies and reviews of the literature. This editorial synthesizes advances across 15 studies, highlighting innovative diagnostic and therapeutic strategies in gastrointestinal oncology. Case studies emphasize targeted approaches for rare cancers, such as SMARCA4-deficient tumors and metastatic hepatocellular carcinoma, underscoring precision medicine’s role. Some of the original researches unveils promising biomarkers like cuproptosis and angiogenesis markers in colorectal cancer, methylation subtypes predicting immunotherapy response, and novel imaging techniques distinguishing gastric tumors. Studies on socioeconomic factors reveals organized screening’s role in reducing gastric cancer disparities. A systematic review of guanylate cyclase-C signaling suggests new colorectal cancer therapies. Together, these research papers advance precision oncology and health equity in gastrointestinal cancers (**Figure 1**).

Liang et al. reviews the efficacy and safety of zolbetuximab, a monoclonal antibody targeting Claudin 18.2, for first-line treatment of advanced gastric and gastro-esophageal junction (G/GEJ) adenocarcinoma. A meta-analysis of three randomized controlled trials involving 1,402 patients found that zolbetuximab combined with chemotherapy significantly improved overall survival and progression-free survival compared to chemotherapy alone, especially in patients with high Claudin 18.2 expression. Although associated with more grade 3+ adverse events, primarily nausea and vomiting, these side effects were generally manageable. This combination therapy offers a promising new treatment option for CLDN18.2-positive G/GEJ adenocarcinoma.

Piroozkhah et al. systematically reviews the guanylate cyclase-C (GC-C) signaling pathway’s role in colorectal cancer (CRC) as a potential target for diagnosis, prognosis, and therapy. The GC-C receptor, activated by endogenous peptides guanylin and uroguanylin, regulates intestinal fluid homeostasis and may influence CRC development. Disruption of GC-C signaling is linked to tumorigenesis and reduced cell differentiation. Targeting GC-C through novel treatments, such as vaccines and chimeric antigen receptors, shows promise in preclinical trials. The study underscores GC-C as a significant therapeutic and diagnostic marker, proposing it as a viable target for future CRC treatments.

Zhou et al. reports two cases of gastrointestinal neuroendocrine carcinoma (GI NEC) with SMARCA4 deficiency, a rare genetic alteration associated with aggressive cancer behavior. Both cases, one in the duodenal papilla and the other in the stomach, presented as poorly differentiated tumors with high proliferation indexes. Despite receiving chemotherapy and anti-PD-1 immunotherapy, the outcomes were poor: one patient died within nine months, while the other showed no disease evidence for ten months post-surgery. SMARCA4 deficiency, marked by loss of BRG1 expression, emerges as a potential prognostic and therapeutic biomarker, warranting further investigation.

Li et al. in their study investigates colorectal cancer (CRC) recurrence prediction through a model based on cuproptosis and angiogenesis markers. Using long non-coding RNAs (lncRNAs) related to copper-induced cell death (cuproptosis) and angiogenesis, researchers developed a Cox regression model to identify high-risk CRC patients for recurrence post-surgery. Results showed strong prediction accuracy, validated across different datasets. Additionally, the study highlights a negative correlation between cuproptosis and angiogenesis, supported by differential immune cell compositions in high- and low-risk groups. This model potentially aids in personalized therapy, identifying patients likely to benefit from more intensive treatment strategies.

The corrigendum by Du et al. addresses a funding statement error in a previous article on cardiac tamponade, a rare complication following gastric cardia cancer resection combined with neoadjuvant chemotherapy and immunotherapy. The corrected funding source is “Key Projects of Medical Science Research of Hebei Province (20230795)” rather than “(2023079).” This amendment does not affect the scientific conclusions or findings discussed in the original publication.

Lin et al. presents two cases of SMARCA4-deficient gastric carcinoma, a rare and aggressive cancer variant marked by the loss of SMARCA4 expression. Both cases exhibited poor differentiation with distinct histological characteristics, including rhabdoid cells, and showed poor response to conventional therapies. A review of 29 reported cases emphasizes high mortality rates and poor prognosis, with a median survival of eight months. The study categorizes SMARCA4-deficient gastric carcinomas into three subtypes based on histological features and immunophenotype, advocating for precise diagnosis and potentially exploring targeted therapies like EZH2 inhibitors for these challenging cases.

Chen et al. by their study investigates the prognostic value of preoperative plasma fibrinogen (PF) and C-reactive protein/albumin (CRP/Alb) ratio in pancreatic carcinoma patients. Analyzing 250 patients, it finds that higher PF and CRP/Alb levels correlate with poor overall survival (OS) and advanced clinical stages. Particularly, in patients with pancreatic ductal adenocarcinoma (PDAC), combining high PF and CRP/Alb levels serves as a stronger predictor of OS compared to each marker alone. The findings suggest that the PF and CRP/Alb ratio could help identify high-risk patients who might benefit from more aggressive treatment strategies.

Wojakowska et al. explores proteomic and metabolomic markers in rectal cancer to predict patient response to preoperative radiotherapy (neo-RT). By analyzing tissue samples, researchers identified proteins and metabolites associated with good and poor responses to treatment. Key differences were found in energy metabolism, amino acid degradation, and immune-related pathways. Proteins like CEACAM5 and metabolic shifts, such as elevated glycolysis in poor responders, were highlighted. The findings suggest that multi-omic panels can serve as biomarkers to predict and monitor radiotherapy efficacy, potentially guiding personalized treatment strategies for rectal cancer patients.

One of the researches made by Cao et al. studies a randomized controlled trial protocol for using the Yiqi Wenyang Jiedu prescription (YWJP), a traditional Chinese medicine (TCM) formula, to prevent recurrence and metastasis in gastric cancer (GC) patients post-surgery. Involving 212 patients who completed adjuvant chemotherapy, the trial will assess the effectiveness of YWJP in enhancing disease-free survival (DFS) compared to a placebo. Secondary outcomes include overall survival, recurrence rates, quality of life, and adverse reactions. This study aims to provide robust evidence for YWJP’s role in GC management, potentially aiding in the development of integrative postoperative therapies.

Xu et al. identifies DNA methylation-driven subtypes of colon cancer to predict immunotherapy responsiveness. Using multi-omics data, researchers identified a specific cluster (Cluster 1) with distinct immune characteristics, such as high tumor mutational burden (TMB), CD8+ T-cell infiltration, and immune checkpoint expression, suggesting better outcomes with immunotherapy. The study also highlights three methylation markers—PCDH20, APCDD1, and COCH—as effective indicators for this immunotherapy-beneficial subtype. Validation showed these markers’ strong predictive capability, supporting their use in expanding immunotherapy eligibility beyond microsatellite instability status. The findings provide a promising approach for precision immunotherapy in colon cancer.

Liu et al. in their case report describes a rare malignant glomus tumor of the esophagus with metastases to the lungs and liver in a 49-year-old patient. The treatment combined Anlotinib, a multi-target anti-angiogenic agent, with Tislelizumab, an immunotherapy drug, based on genetic testing. This personalized approach achieved significant clinical improvement, including tumor size reduction and improved quality of life. However, the disease ultimately progressed, leading to the patient’s death due to gastrointestinal bleeding. This case underscores the potential of precision medicine, especially the role of next-generation sequencing, in treating rare malignancies with targeted therapies.

Zhao et al. in their retrospective study developed a diagnostic model to differentiate gastric schwannomas from gastrointestinal stromal tumors (GISTs) using computed tomography (CT) imaging features. A nomogram was constructed based on factors such as growth pattern, absence of necrosis, presence of tumor-associated lymph nodes, and contrast differences across CT phases. The model, validated in multiple centers, demonstrated high diagnostic accuracy with an area under the curve (AUC) of 0.937 in the training set and 0.921 in the validation set. This non-invasive tool supports accurate preoperative identification, helping inform surgical decisions and improve patient.

Esmaeili et al. identifies the role of RNA modification, specifically N6-methyladenosine (m6A), in the progression of chronic inflammation-induced colorectal cancer (CRC) via the JAK-STAT3 and STAT5 signaling pathways. m6A modification impacts RNA stability, splicing, and translation, influencing gene expression linked to cancer cell proliferation and metastasis. Dysregulation of m6A and STAT pathways is shown to promote CRC by altering inflammatory responses, immune regulation, and epithelial integrity. The study emphasizes m6A’s potential as a therapeutic target, proposing that targeting m6A and JAK-STAT interactions could offer new avenues for CRC treatment.

The last case report by Cui et al. discusses a patient with advanced hepatocellular carcinoma (HCC) and extrahepatic metastasis achieving complete remission through a combination of sintilimab (an immune checkpoint inhibitor) and sorafenib (an anti-angiogenic agent), followed by liver resection. After six cycles of the combination therapy, significant tumor reduction and metastasis resolution were observed, enabling surgical resection. Post-surgery, the patient has remained disease-free for over two years. This case suggests that combining immunotherapy and targeted therapy could downstage advanced HCC to allow curative resection, potentially leading to prolonged survival even in metastatic cases.

Luu et al. analyzes socioeconomic inequalities in gastric cancer (GC) screening in Korea, using data from the Korean National Cancer Screening Survey (2009–2022). Organized GC screening rates increased significantly, from 38.2% in 2009 to 70.8% in 2022, while opportunistic screening rates declined. Socioeconomic disparities were minimal in organized screenings, indicating equitable access across different income and education levels. However, substantial inequalities persisted in opportunistic screenings, with higher rates among wealthier and more educated individuals. The findings emphasize the role of organized screening programs in reducing inequalities and improving cancer screening accessibility across socioeconomic groups.

Our Research Topic highlights significant strides in precision oncology. Through innovative biomarkers, genetic profiling, and targeted therapies, these studies illustrate how molecular insights are reshaping diagnosis, treatment, and screening strategies, particularly for challenging cases like rare SMARCA4-deficient cancers and advanced-stage hepatocellular carcinoma. Research on socioeconomic disparities in gastric cancer screening also reveals the importance of equitable healthcare access, aligning molecular advancements with public health goals. Collectively, this Research Topic reinforces the critical role of molecular targets in transforming gastrointestinal cancer care toward personalized and accessible approaches by publishing 15 extremely valuable articles.

